# An experimental *Staphylococcus aureus* carriage and decolonization model in rhesus macaques (*Macaca mulatta*)

**DOI:** 10.1371/journal.pone.0194718

**Published:** 2018-04-12

**Authors:** Bibi C. G. C. Slingerland, Merei Keehnen, Boudewijn Ouwerling, Mehri Tavakol, Susan V. Snijders, Henri A. Verbrugh, Margreet C. Vos, Edmond J. Remarque, Jan A. M. Langermans, Willem J. B. van Wamel

**Affiliations:** 1 Department of Medical Microbiology and Infectious Diseases, Erasmus MC, Rotterdam, The Netherlands; 2 Animal Science Department, Biomedical Primate Research Centre, Rijswijk, The Netherlands; 3 Department of Virology, Biomedical Primate Research Centre, Rijswijk, The Netherlands; University of Central Florida College of Medicine, UNITED STATES

## Abstract

Our human model of nasal colonization and eradication of *S*. *aureus* is limited by safety issues. As rhesus macaques are closely related to humans and natural hosts for *S*. *aureus*, we developed an experimental decolonization and inoculation protocol in these animals. Animals were screened for nasal carriage of *S*. *aureus* and 20 carriers were selected. Decolonization was attempted using nasal mupirocin (10 animals) or mupirocin plus trimethoprim/sulfadiazine intramuscularly (10 animals) both once daily for 5 days, and checked by follow-up cultures for 10 weeks. Intranasal inoculation was performed with *S*. *aureus* strain 8325–4 in culture-negative animals. 11/20 animals, of which 5 received mupirocin and 6 the combination treatment, became culture-negative for *S*. *aureus* for 10 weeks and these 11 animals were subsequently inoculated. Swabs were taken once a week for 5 weeks to test for the presence of the inoculated strain. In 3 animals, strain 8325–4 was cultured from the nose 1 week after inoculation, indicating short-term survival of this strain only, a finding similar to that previously found in our human model. These data demonstrate that rhesus macaques may constitute a relevant animal model to perform *S*. *aureus* eradication and inoculation studies with relatively limited invasive handling of the animals.

## Introduction

It is well established that *Staphylococcus aureus* (*S*. *aureus*) carriage increases the risk of endogenous infections with this bacterium [1, 2]. Bode et al. showed that eradication of *S*. *aureus* from the nose and skin significantly decreases the risk of endogenous infections in a hospital setting [3]. Many different (methicillin-resistant and susceptible) *S*. *aureus* eradication and decolonization strategies have been described. Variations in these treatments are made based on the presence of clinically evident infection [4]. To further improve our understanding of human *S*. *aureus* nasal colonization and to support the development of new decolonization strategies additional well-designed experiments are needed. Although we have performed human nasal inoculation studies in our group [5–9], for ethical and safety reasons these experiments are restricted to a conformational setup while more exploratory designs are often desired. Therefore, animal models of *S*. *aureus* carriage are needed and some have been presented in the literature. Ideally, an animal model closely mimics the human condition, in this case requiring the animal to be a natural host of *S*. *aureus* and to be evolutionary closely related to humans. Furthermore, (de) colonization is best studied over a period of time in which at different time points of follow-up samples can be taken from the same animal. In one model the cotton rat requires relatively high inocula (1*10^7^ to 1*10^9^ CFU) in order to establish nasal colonization [10–12]. In addition, animals need to be sacrificed at pre-specified intervals in order to determine bacterial colonization. Thus, bacterial and host factors interfering with colonization cannot be studied longitudinally as for each time-point separate groups of animals are included [10–12]. The same limitations apply for mouse models [13, 14]. Successful *S*. *aureus* colonization has been realized in pigs, but only in piglets delivered by Caesarean section and deprived of colostrum [15, 16]. In addition to research regarding determinants of colonization, *S*. *aureus* decolonization strategies have also been studied in mice and cotton rats, but we are confronted with the same limitations [17–19]. Recently, rhesus macaques (*Macaca mulatta*) were added to the list of natural hosts of *S*. *aureus* [20] making them potentially an ideal human-like model to study *S*. *aureus* (de) colonization.

Therefore, in this study we explored the possibility to develop an experimental decolonization and inoculation procedure in rhesus macaques. To do so, we applied two decolonization strategies; both used in humans, and investigated their potential to decolonize the nares of rhesus macaques carrying *S*. *aureus* in their nares. In addition, we studied the possibility to colonize rhesus macaques with a human *S*. *aureus* strain.

## Materials and methods

### Study setup

Rhesus macaques were screened for *S*. *aureus* nasal carriage and carriers were included (definition below). The study design had two phases: in phase 1 *S*. *aureus* decolonization with two different treatment strategies was performed, followed by phase 2 in which nasal inoculation with a human *S*. *aureus* strain was attempted.

### Animal study population

Animals were selected from the macaque-breeding colony of the Biomedical Primate Research Centre Rijswijk, the Netherlands. They were born and raised at the institute and remained in their natal groups for at least 4 years before being selected for research protocols. All animals received a complete physical, haematological and biochemical examination before inclusion and were social-housed (pair or trio) for at least 3 weeks before the onset in 3 animal rooms. Physical contact with other rhesus macaques from the same experimental group in neighboring cages was possible. In each room the cages were located in two rows opposite to each other. Because of welfare some of the animals were placed with their buddy rhesus macaque during phase 1 and 2. A buddy rhesus macaque was not screened for *S*. *aureus* nasal carriage but it did receive the same decolonization treatment as its *S*. *aureus* carrier cage mate. Their culture results were not included in the study.

### Ethical considerations

All animals were socially housed in cages measuring at least 3.6 m^3^ (2x1.8x1 m) in line with the size requirements of EU Directive 2010/63. Cages were provided with bedding and food and non-food enrichment items. Water was provided *ad libitum*. The animals were fed commercial monkey pellets (Ssniff, Soest, Germany) supplemented with a variation of bread, vegetables and fruit. All experimental procedures with the animals were performed under sedation with 10-mg/kg ketamine. The study protocol was approved by the Animal Ethics Committee of the Biomedical Primate Research Centre (BPRC) Rijswijk, The Netherlands (DEC #706).

### Sampling procedures

The nose was sampled by swabbing both the left and right anterior nares with a single swab (Amies charcoal transport swabs orange, Copan, CA, USA). Throat and rectal swabs (Amies charcoal transport swabs black, Copan, CA, USA) were taken respectively by swabbing the back of the pharynx and by revolving a swab 2 cm into the rectum. The swabs were plated on Colombia 5% sheep blood agar (OXOID, Hampshire, UK) within a few hours. Plates were screened for *S*. *aureus* suspected colonies based on morphology after 24 and 48 hours of incubation at 35°C. Final identification of *S*. *aureus* was performed by a latex slide agglutination test that detects clumping factor and Protein A (Staphytect Plus, OXOID, Hampshire, UK).

### Susceptibility of the *S*. *aureus* strains isolated from the rhesus macaques

The VITEK 2 system (bioMérieux) was used for susceptibility testing. The *S*. *aureus* strains were picked randomly and had to originate from the nose from day 0 of the decolonization phase. *S*. *aureus* colonies were suspended in 0.9% saline (NaCl) to make a 0.5 McFarland (McF) bacterial suspension. Susceptibility card AST-P633 was used for staphylococci. This card contained benzylpenicillin, oxacillin, gentamicin, kanamycin, tobramycin, ciprofloxacin translated to levofloxacin, erythromycin, clindamycin, linezolid, teicoplanin, vancomycin, tetracycline, fosfomycin, fusidic acid, mupirocin, chloramphenicol, rifampicin and trimethoprim/sulfamethoxazole.

### *S*. *aureus* strain for inoculation

For the inoculation procedure we selected human strain 8325–4 (*spa* type 211; ST8) [21] which was previously used in human nasal inoculation studies [5, 8]. Bacteria grown overnight on Colombia 5% sheep blood agar were used to inoculate Brain Heart Infusion broth (BHI). After a few hours of growth at 37°C an OD of 0.5 McF was achieved. The pellet of bacterial culture was suspended in Phosphate Buffered Saline (PBS) to get the inoculum of 2*10^8^ CFU/ml. 50 microliters of this amount of bacteria in PBS contained 1*10^7^ CFU of bacterial cells.

### Screening and decolonization protocol

Taking 4–5 nasal swabs with intervals of one week, determined the nasal carriage state for *S*. *aureus*. Nasal carriers of *S*. *aureus* were defined as animals with four swabs (≥80%) culture-positive for *S*. *aureus*. Only carriers were included. Two different treatment strategies were used for decolonization. One treatment consisted of mupirocin nasal ointment (2%; Glaxo- SmithKline, Waltham, MA, USA) once daily for a total period of 5 days (treatment A). The other treatment consisted of a combination of mupirocin nasal ointment once daily with 18 mg/kg trimethoprim/sulfadiazine (40 mg/ml/200 mg/ml; Duphatroxim, Zoetis, NL) intramuscular injections once daily for a total period of 5 days (treatment B). The mupirocin nasal ointment was applied in the vestibulum nasi with a swab (Heinz Herenz sterile cotton swab). Trimethoprim/sulfadiazine was injected alternately into the left and right vastus lateralis. The study was performed in two different time periods in which the animals were equally divided over the two treatment regimens. In the follow-up period of 9 weeks a total of 9 (including t = 0, before start of the treatment) nasal, throat and rectal swabs were taken to investigate recolonization with *S*. *aureus*. More specifically, on day 0, 3 or 4, 7, 9, 14, 21, 35, 49 or 50 and 63 swabs were taken from the three culture sites.

### Inoculation protocol

Nasal inoculation was only performed in animals successfully decolonized. Noses of animals were inoculated 7 days after the last follow-up culture moment from phase 1. As *S*. *aureus* nasal carriage was an exclusion criterion for further follow-up after inoculation, a control swab was taken from all culture sites on the day of inoculation. 50 microliters of the inoculum containing 1*10^7^ CFU of bacterial cells was pipetted separately into the left and right nostril while the head was put for two minutes in such a position that the inoculum could be absorbed by the mucosa. In the follow-up period the nose, throat and rectum were cultured in week 1, 2, 3 and 5. For identification of strain 8325–4 after inoculation, all cultured *S*. *aureus* strains obtained in the follow-up period from the inoculation phase, were analyzed by PCR analysis of the *spa* gene [22].

### Hygiene protocol

To prevent any *S*. *aureus* acquisition from environmental, human or animal origin during both phases of the study, a hygiene protocol was implemented. Before entering the animal room personnel had to wear non-sterile gloves (Kimtech) and a new gown including a hood (Tyvek). After entering the room gloves were immediately disinfected with alcohol 70%. Care was taken to handle first the animals included in the study to prevent possible spread of *S*. *aureus* from animals that were not included. During the five consecutive days of sedation in the decolonization phase, the animals received 100 ml Nutrison Concentrated Food 2.0 kcal/ml through a gastric tube to prevent malnutrition. Non-food enrichment was cleaned with alcohol before rotation and bedding was replaced twice a week. For activities like contact with every separate cage animal caretakers disinfected their hands. Finally, keys were put in a bath with alcohol 70% to limit contamination.

### Statistical analysis

Recurrence, defined as the first occasion when *S*. *aureus* was isolated again after decolonization, was analyzed starting from day 7 after start of the decolonization. Recurrence was determined in either nose only, other sites than the nose or any site, i.e. nose, throat and/or rectum. Results were analyzed using Kaplan-Meier curves.

## Results

### Screening and decolonization

A total of 97 rhesus macaques were selected from the BPRC colony and their *S*. *aureus* carriage status was determined. 20 out of 73 rhesus macaques that were at least once positive for *S*. *aureus* in the nose were found to be nasal *S*. *aureus* carriers. In total 9 buddies participated. Divided over 13 cages, 3 animals were triple-housed and the others were pair-housed. 10 rhesus macaques received treatment A (3 females, 7 males) and the others received treatment B (3 females, 7 males). Follow-up cultures were taken from the nose, throat and rectum.

Individual culture results from the start of both treatments including the follow-up period are shown in S1 Table. In the group that received treatment A, 2 animals (R10024 and R10122) were negative in the nose at day 0 and they were therefore excluded from analysis. For completeness, culture results from the buddy rhesus macaques were analyzed and added in this table. The results show in most cases variable carriage rates on the three culture sites in the different animals during follow-up. No specific carriage patterns were seen in the individual rhesus macaques except for some animals in which throat carriage was more persistent over time than in the other animals. Furthermore, cage mates were seldom carrier at the same culture site at the same culture moment (S1 Table).

In animals that received mupirocin only (treatment A) no *S*. *aureus* was cultured from the nose in any of the animals from day 7–21. *S*. *aureus* was observed in one animal after 21 days but all animals were once more negative in the following culture moment at day 35. From day 49 after the treatment 2 animals were again colonized with *S*. *aureus* in the nose (S1A Fig). In this treatment group a rapid decline in *S*. *aureus* throat and rectum carriage to 0% was observed within the first nine days. In some animals recolonization occurred from day 14 until the end of the follow-up period (S1 Table, S1B Fig).

*S*. *aureus* was eradicated from the noses of all animals that received mupirocin and trimethoprim/sulfadiazine (treatment B) from day 7–21 after the start of treatment. On day 35 and 49, 2 animals were positive in the nose for *S*. *aureus* and this number increased to 4 at the end of the follow-up period (S1A Fig). Throat carriage rates after treatment B declined from 7 animals to 1 in the first 9 days. In the remaining follow-up period *S*. *aureus* throat carriage varied between 3 and 4 animals. Finally, at the start of treatment B, 1 animal was positive in the rectum for *S*. *aureus* and during the follow-up carriage on this site was at most found in 1 animal (S1 Table, S1B Fig).

**Fig 1:**
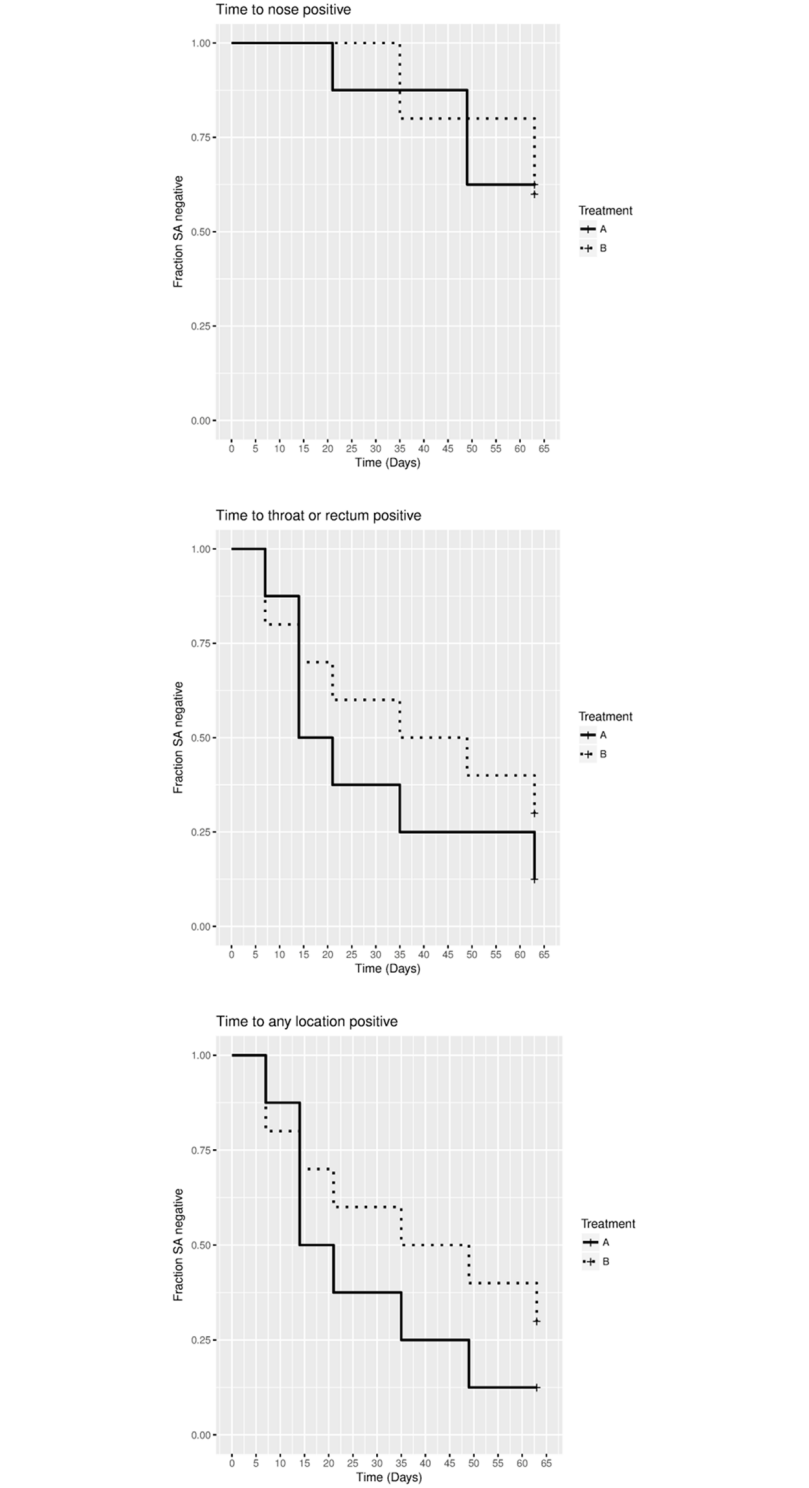
Kaplan-Meier curves. The proportion of *S*. *aureus* positive animals is represented by a continuous (treatment A) or dotted (treatment B) line. Fig 1A, 1B and 1C show respectively results from nose only, other sites than nose and any site.

There was no difference between the treatment groups when the portion of animals positive for *S*. *aureus* was determined for nose only, other sites than the nose or any site. In addition, the first occasion when *S*. *aureus* was cultured after decolonization was analyzed using Kaplan-Meier curves for either nose only (Fig 1A), other sites than the nose (Fig 1B) or any site, i.e. nose, throat and/or rectum (Fig 1C). The data demonstrate that there is no difference between the two treatments with respect to the time that in some animals the nose became positive. Even after 63 days 60% of all animals from both treatment groups were negative in the nose (Fig 1A). However, when throat or rectum were analyzed separately or any site, more animals became positive again and a trend was seen suggesting that in the animals that received treatment B the time to become positive was longer than in animals that received treatment A (Fig 1B and 1C). This trend is not statistically significant because this study concerned a proof-of-principle study for decolonization and it was not powered for the statistical detection of this difference.

### Inoculation

A total of 11 animals never became positive for *S*. *aureus* in the nose during the follow-up period of 63 days (S1 Table). Of these 11 animals, 5 had received treatment A and 6 had received treatment B. Animals were inoculated with human *S*. *aureus* strain 8325–4. 2 rhesus macaques were found to be positive for *S*. *aureus* in the nose on the day of inoculation and they were excluded from further analyses. Follow-up cultures were taken from nose, throat and rectum in week 1, 2, 3 and 5. In 3/9 animals remaining in the experiment, the inoculated *S*. *aureus* strain 8325–4, was found in the nose one week after inoculation. 2 of these animals had received treatment B and the other animal treatment A. The successive follow-up cultures on all culture sites became negative in these 3 animals. In the other 6 animals strain 8325–4 was never cultured but in 3 of them *S*. *aureus* other than 8325–4 was cultured in the throat and in 1 also in the rectum.

### Susceptibility of the *S*. *aureus* strains isolated from the rhesus macaques

For susceptibility testing 10 *S*. *aureus* strains were used. These strains were derived from the nasal cavities of 10 different animals. All strains were susceptible to all the antimicrobials that were tested, including mupirocin and trimethoprim/sulfamethoxazole.

## Discussion

To our knowledge this is the first study in which a controlled experimental *S*. *aureus* nasal decolonization and inoculation model in rhesus macaques is presented. With the standardized protocols the noses of carriers were successfully decolonized for a period of 10 weeks or more. We tested 2 *S*. *aureus* decolonization strategies, a topical treatment with mupirocin nasal ointment and a combination treatment of mupirocin nasal ointment with systemic trimethoprim/sulfadiazine. There was no significant difference between the two treatments as almost all animals became negative for *S*. *aureus* in the nose, throat and rectum and in both groups comparable numbers of animals became positive again. Also with respect to time of first recurrence of *S*. *aureus*, there was no difference between the two treatment groups when only the nose was analyzed. However, analysis of recurrence of *S*. *aureus* at any site showed a trend suggesting that treatment B was more effective than treatment A.

The two different treatment strategies for decolonization of nasal carriers were based on the eradication strategies for complicated and uncomplicated MRSA carriage in humans. Uncomplicated carriage implies nasal colonization only and complicated carriage is defined as nasal colonization in combination with one or more of the following criteria: extra-nasal carriage sites, or a clinically active infection, or the presence of skin lesions, foreign-body material, or the finding of mupirocin resistance in the colonizing strain. For uncomplicated carriage mupirocin nasal ointment and chlorhexidine-containing body wash is recommended for humans. In complicated *S*. *aureus* carriers this topical treatment regimen is augmented with systemic antimicrobials [4].

In both protocols used in this study we did not add chlorhexidine-containing body washes because of two practical reasons: the large amount of body hair of these animals was thought to limit the effectiveness of skin decolonization and body washes would require additional periods of anesthesia which would increase the risk of complications. A systemic intramuscularly injectable antimicrobial trimethoprim/sulfadiazine was chosen because it is registered in veterinary medicine in the Netherlands whereas cotrimoxazole (trimethoprim/sulfamethoxazole), commonly used in human medicine, does not guarantee optimal oral uptake in animals. Furthermore, Ammerlaan *et al*. recommended to always using a combination of two systemic antimicrobials to eradicate *S*. *aureus*, and to choose an antimicrobial combination that included either fusidic acid or rifampicin [4]. However, these latter two antimicrobials are not registered in veterinary medicine and were therefore, not considered for this series of experiments. In order to limit the number of sedations we administered both treatments once daily. Our data indicate that (un) complicated *S*. *aureus* carriage in rhesus macaques can well be eradicated with topical applied mupirocin alone, the addition of systemic trimethoprim/sulfadiazine did not enhance decolonization.

Although we were able to inoculate the noses of the rhesus macaques with human *S*. *aureus* strain 8325–4, we did not observe stable long-term colonization with this strain. Though, in previous studies, *S*. *aureus* 8325–4 was shown to persist for at least 4 weeks in the human nose despite the presence of a defect in the sigma factor (SigB) locus [5, 8, 23]. By choosing this strain we had the benefit to be able to use our previous experience in human inoculation experiments as a starting point. Furthermore, 8325–4 is suitable for genetic modification and can be applied for both knockout [5, 8] and knock-in studies. On the contrary, by selecting 8325–4 we took the risk on poor colonization, yet we wanted to investigate if we could use a human strain to inoculate a rhesus macaque. Our data show that 8325–4 presumably is not the ideal strain to work with in the rhesus macaque model. In a preceding study we have shown that rhesus macaques are naturally colonized with *S*. *aureus* strains that have genetically different backgrounds from human strains of this species [20]. Therefore, in future experiments, selecting a strain from the collection of *S*. *aureus* cultured from rhesus macaque (persistent) carriers might show to be a better take.

In addition to natural variation in nasal microflora, an alternative explanation for our limited success in the colonization phase of the study could be the method we used to apply the inoculum. In earlier human inoculation experiments 1*10^7^ CFU were applied in the nose with a swab [5–9] but the (smaller) nasal anatomy of rhesus macaques does not allow to use the same swab as used in humans. Therefore, we had to adapt the inoculation procedure, which might have resulted in loss of part of the bacterial inoculum due to swallowing. Although, in the cotton rat model the same inocula and method of instillation are used for nasal inoculation experiments, in the rat it seems more successful than in our rhesus macaque model in comparable periods of follow-up. Possibly, this is due to the fact that, after sacrificing the rats, noses are completely removed and then homogenized which might result in better recovery of the bacteria from the nose [10–12]. A third possible explanation could be that mupirocin was still active at the time of inoculation. Fernandez et al. studied the efficacy of mupirocin on *S*. *aureus* nasal colonization in which recolonization after 2–4 months was seen in 56% of the study population [24]. On the contrary, our human model [5–9] showed successful inoculation 6 weeks after decolonization with mupirocin and chlorhexidine-containing body wash. Nevertheless, we showed that we were able to inoculate the noses of some of the rhesus macaques for at least one week.

Concerning nasal *S*. *aureus* carriage, animals were swabbed 4–5 times with intervals of one week. We defined carriage as animals in which at least 80% of the nasal cultures had to be *S*. *aureus* positive. Our definition is in line with the definition of (persistent) carriage used in several publications of human studies. However, in some publications additional criteria are defined which are related to the number of consecutive swabs that have to be taken, the number of CFU/swab but also the added value to test for genetic background [9, 25–28]. While many variations in definition exist, for this study we elected to stick to one that is practical and commonly used in literature.

During the development of this model we were confronted with a couple of issues. Two animals were culture-negative for *S*. *aureus* in the nose on the day of start of the treatments. These animals were already classified as nasal carriers, so this result required exclusion. It is apparent that in macaques, as in humans, carriage rate and densities, although we did not culture quantitatively, are prone to some variation over time, which was also seen in the follow-up period after treatment A and B, especially for the extra-nasal culture sites. We observed very low carriage rates in the rectum, which could be a result of our culture conditions. *S*. *aureus* could have been overgrown by gram negative bacteria from the gut so maybe a selective agar would have been a better choice. Another, the throat in particular, was alternately positive in most of the animals. Recognition of this phenomenon should be acknowledged when designing, implementing and interpreting studies on decolonization.

Another finding, albeit not a focus in this study, concerned the transmission of *S*. *aureus*. Mollema et al. showed that (human) household members have a risk of 47% to transmit *S*. *aureus* when one of them is positive [29]. In our study we showed that some monkeys including the buddies never acquired any *S*. *aureus* in the follow-up period while *S*. *aureus* was present in their cage mate. Also, some animals were positive for *S*. *aureus* at a specific carrier site while the cage mate was a carrier at another site. Since we did not type these strains it is unknown whether transmission of *S*. *aureus* had occurred or not. Finally, we used *spa* typing, which is less delicate than for example whole genome sequencing but it served its purpose of identifying strain 8325–4.

In conclusion, this model can be used for *S*. *aureus* decolonization and inoculation studies in a properly controlled fashion. In contrast to small animal models, this model is attractive since this species of animals allows for long-term follow-up of the carrier state in individual subjects, and does not require animals to be sacrificed as part of the experimental protocol. Moreover, macaques are natural carriers of *S*. *aureus* and are closely related to humans, which makes them also attractive to study host-pathogen interaction.

## Supporting information

S1 Table *S*. *aureus* carriage in nose, throat and rectum in the individual rhesus macaque and their cage buddies in the follow-up period of the decolonization phase. (DOCX)

S1 Fig *S*. *aureus* decolonization of rhesus macaques. Each red or blue dot represents the fraction of cultures positive for *S*. *aureus* before and after treatment A (mupirocin; red) and B (mupirocin and trimethoprim/sulfadiazine; blue). Nasal carriage and carriage at any site are shown in S1A and S1B Fig respectively.
(DOCX)
