# Supplementary material for: An experimental Staphylococcus aureus carriage and decolonization model in rhesus macaques (Macaca mulatta)
Source: PLoS One. 2018 Apr 12;13(4):e0194718. doi: 10.1371/journal.pone.0194718 (PMC5896908; doi:10.1371/journal.pone.0194718)
Supplement: S1 Table — (DOCX) [file pone.0194718.s001.docx]

**Supporting Information**

**S1 Table**

| **Room** | **Cage** | **Animal** | **Treatment** | **Day 0** | **Day 3/4** | **Day 7** | **Day 9** | **Day 14** | **Day 21** | **Day 35** | **Day 49/50** | **Day 63** |
| --- | --- | --- | --- | --- | --- | --- | --- | --- | --- | --- | --- | --- |
| **43** | **3** | **R09147** | **A** | **N/T +** | **-** | **-** | **-** | **-** | **T +** | **-** | **-** | **-** |
|  |  | **R11080** | **A** | **N +** | **-** | **-** | **-** | **-** | **-** | **-** | **-** | **-** |
| **43** | **5** | **R10034** | **A** | **N/T/R+** | **N +** | **-** | **-** | **-** | **-** | **T +** | **T +** | **-** |
|  |  | **R11098** | **A** | **T/R +** | **-** | **-** | **-** | **T +** | **T +** | **-** | **-** | **-** |
| **45** | **1** | **R10024** | **A** |  |  | | | | | | | |
|  |  | **R04062** | **A** | **N/T +** | **-** | **-** | **-** | **T +** | **-** | **-** | **T +** | **-** |
| **45** | **3** | **R05070** | **A** | **N/T +** | **-** | **-** | **-** | **R +** | **-** | **-** | **-** | **-** |
|  | ***** | **R07022** | **A** | **N +** | **-** | **-** | **-** |  | | | | |
| **45** | **6** | **R07076** | **A** | **N/T/R+** | **T +** | **T +** | **-** | **-** | **T +** | **-** | **N/T +** | **N +** |
|  |  | **R08133** | **A** | **N/T +** | **-** | **-** | **-** | **-** | **-** | **-** | **-** | **-** |
| **46** | **1** | **R10122** | **A** |  |  | | | | | | | |
|  |  | **R02038** | **A** | **-** | **-** | **-** | **-** | **-** | **T +** | **-** | **-** | **-** |
| **46** | **2** | **R07026** | **A** | **N/T +** | **-** | **-** | **-** | **-** | **-** | **-** | **N +** | **T +** |
|  |  | **R08134** | **A** | **-** | **-** | **-** | **-** | **-** | **-** | **-** | **-** | **-** |
| **46** | **3** | **R10030** | **A** | **N/T/R+** | **T +** | **-** | **-** | **T +** | **N/R +** | **T/R +** | **R +** | **N/T +** |
|  |  | **R11057** | **A** | **N/R +** | **T +** | **-** | **-** | **T +** | **-** | **N +** | **N/T/R+** | **N/T +** |
| **43** | **2** | **R10053** | **B** | **N/T +** | **T +** | **T +** | **T +** | **T +** | **T +** | **T +** | **T +** | **T +** |
|  |  | **R11093** | **B** | **N +** | **T +** | **-** | **-** | **-** | **N/T +** | **-** | **T +** | **-** |
| **43** | **4** | **R08055** | **B** | **N/T +** | **T +** | **T +** | **-** | **T +** | **T +** | **N/T +** | **N/T +** | **N/T +** |
|  |  | **R11119** | **B** | **N +** | **-** | **-** | **-** | **-** | **-** | **-** | **-** | **-** |
| **45** | **4** | **98007** | **B** | **N +** | **N +** | **-** | **-** | **-** | **-** | **-** | **-** | **T +** |
|  |  | **98024** | **B** | **N +** | **-** | **-** | **-** | **-** | **-** | **-** | **-** | **-** |
|  |  | **98036** | **B** | **N +** | **-** | **-** | **-** | **-** | **-** | **-** | **-** | **-** |
| **46** | **4** | **R00035** | **B** | **N/T +** | **-** | **-** | **-** | **-** | **-** | **-** | **T +** | **N +** |
|  |  | **R01079** | **B** | **N/T +** | **R +** | **-** | **-** | **-** | **-** | **N/T/R+** | **N/T/R+** | **N +** |
| **46** | **5** | **R10069** | **B** | **N/T/R+** | **T +** | **-** | **-** | **-** | **T +** | **-** | **-** | **-** |
|  |  | **R09041** | **B** | **N/T +** | **-** | **-** | **-** | **-** | **-** | **-** | **-** | **-** |
| **46** | **6** | **R07124** | **B** | **N/T +** | **-** | **-** | **-** | **T +** | **T/R +** | **-** | **-** | **N +** |
|  |  | **R07123** | **B** | **-** | **-** | **-** | **-** | **-** | **-** | **-** | **-** | **N +** |

**^a.^ N = nose; T = throat; R = rectum**

**^b.^ + = positive for *S. aureus*; - = negative for *S. aureus***

**^c.^ Treatment A = mupirocin; Treatment B = mupirocin and trimethoprim/sulfadiazine**

**^d.^ Grey background = buddy**

**^e.^ Yellow background = excluded animals from the decolonization phase**

**^f.^ * = Buddy R07022 was euthanized because of severe complications of diverticulosis.**
