# Supplementary figures and images for: An experimental Staphylococcus aureus carriage and decolonization model in rhesus macaques (Macaca mulatta)
Source: PLoS One. 2018 Apr 12;13(4):e0194718. doi: 10.1371/journal.pone.0194718 (PMC5896908; doi:10.1371/journal.pone.0194718)

**Supporting Information**

**S1 Fig**

**S1A**

**
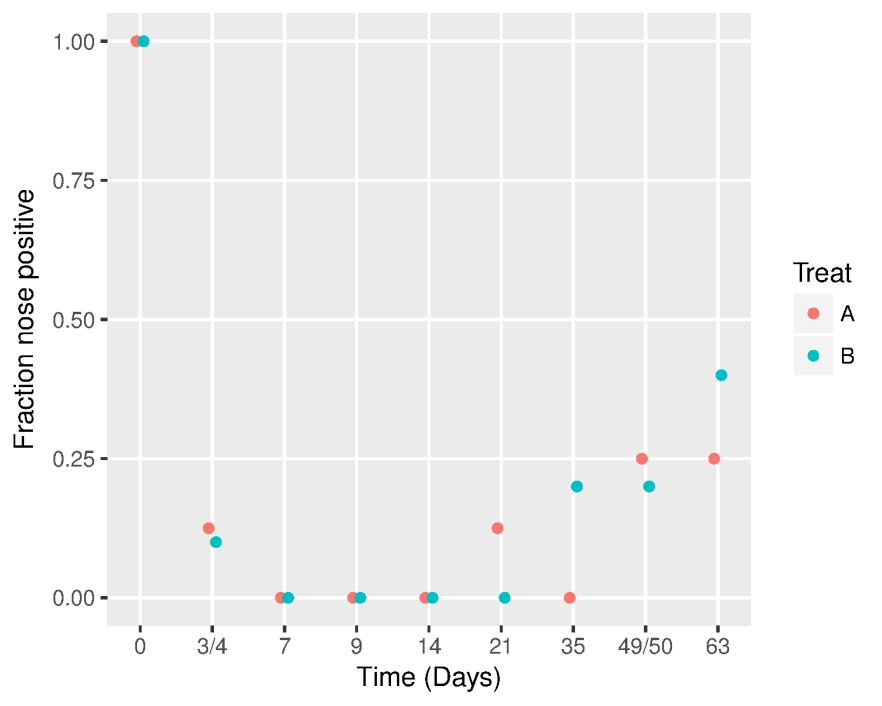
**

**S1B**

**
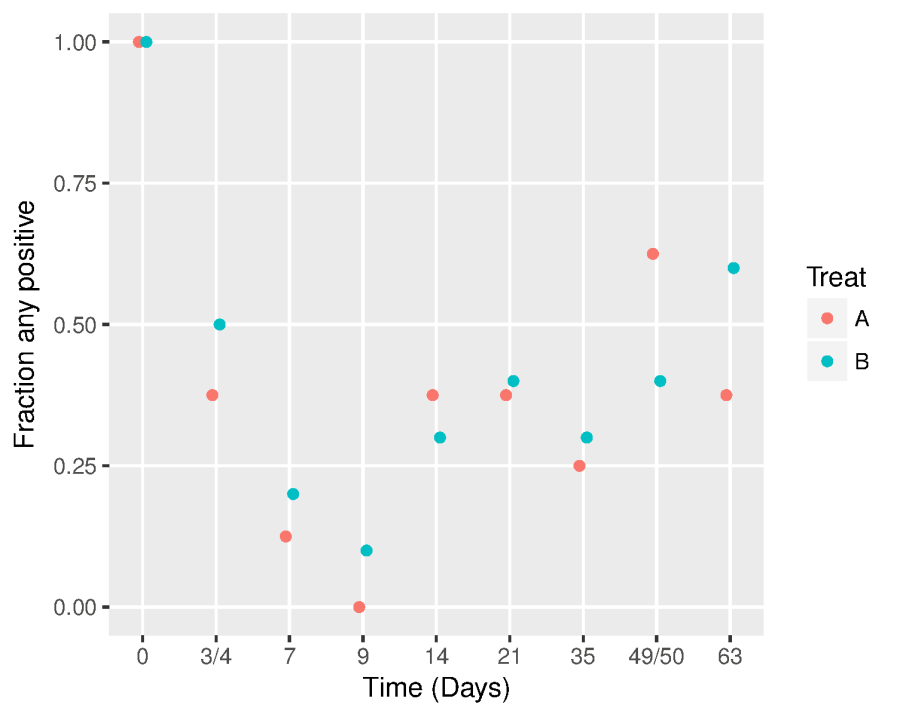
**

Supplement: S1 Fig — Each red or blue dot represents the fraction of cultures positive for S. aureus before and after treatment A (mupirocin; red) and B (mupirocin and trimethoprim/sulfadiazine; blue). Nasal carriage and carriage at any site are shown in S1A and S1B Fig respectively. (DOCX) [file pone.0194718.s002.docx]
